# Supplementary material for: Skeletal variation in bird domestication: limb proportions and sternum in chicken, with comparisons to mallard ducks and Muscovy ducks
Source: PeerJ. 2022 Apr 27;10:e13229. doi: 10.7717/peerj.13229 (PMC9055999; doi:10.7717/peerj.13229)
Supplement: Supplemental Information 2 — Pairwise comparisons were only conducted in breeds with sample size greater or equal to 4. P-values lower than 0.05, considered to reject equality of means, are shown in bold. Na, not applicable. [file peerj-10-13229-s002.docx]

Supplementary File S1. Results of pairwise Wilcoxon rank sum tests to show the differences of limb proportions among the different chicken breeds (see also Table 5 and Fig. 8 in the main manuscript). Pairwise comparisons were only conducted in breeds with sample size greater or equal to 4. P-values lower than 0.05, considered to reject equality of means, are shown in bold. Na = not applicable.


| **Humerus** | | | | | | | | | | | | | | | | | | |
| --- | --- | --- | --- | --- | --- | --- | --- | --- | --- | --- | --- | --- | --- | --- | --- | --- | --- | --- |
|  | | Chabo | Bantam | | Cochin (dwarf) | | Red junglefowl | | | Ukokkei | Polish | | White leghorn | | Appenzeller Spitzhaubenhuhn | | Araucana | Cochin (standard) |
| Bantam | | 0.6483 | NA | | NA | | NA | | | NA | NA | | NA | | NA | | NA | NA |
| Cochin (dwarf) | | 0.4635 | 0.6310 | | NA | | NA | | | NA | NA | | NA | | NA | | NA | NA |
| Red junglefowl | | **0.0000** | **0.0004** | | **0.0244** | | NA | | | NA | NA | | NA | | NA | | NA | NA |
| Ukokkei | | **0.0029** | **0.0277** | | 0.3011 | | 0.3781 | | | NA | NA | | NA | | NA | | NA | NA |
| Polish | | **0.0002** | **0.0020** | | 0.0546 | | **0.0040** | | | **0.0114** | NA | | NA | | NA | | NA | NA |
| White leghorn | | 0.0645 | 0.0989 | | 0.5500 | | 0.7007 | | | 0.6483 | 0.4160 | | NA | | NA | | NA | NA |
| Appenzeller Spitzhaubenhuhn | | **0.0006** | **0.0040** | | 0.0755 | | 0.0517 | | | **0.0364** | 0.4867 | | 0.3274 | | NA | | NA | NA |
| Araucana | | **0.0000** | **0.0001** | | **0.0116** | | **0.0001** | | | **0.0010** | 0.7417 | | 0.0907 | | 0.2718 | | NA | NA |
| Cochin (standard) | | 0.3039 | 0.5256 | | 0.7417 | | 0.7734 | | | 0.7199 | 0.6548 | | 0.8571 | | 0.6687 | | 0.6483 | NA |
| Shamo | | **0.0000** | **0.0006** | | **0.0317** | | **0.0052** | | | **0.0163** | 0.5548 | | 0.4444 | | 0.7007 | | 0.4867 | 0.7901 |
|  | |  |  | |  | |  | | |  |  | |  | |  | |  |  |
| **Radius** | | | | | | | | | | | | | | | | | | |
|  | | Chabo | Bantam | | Cochin (dwarf) | | Red junglefowl | | | Ukokkei | Polish | | White leghorn | | Appenzeller Spitzhaubenhuhn | | Araucana | Cochin (standard) |
| Bantam | | 0.8949 | NA | | NA | | NA | | | NA | NA | | NA | | NA | | NA | NA |
| Cochin (dwarf) | | 0.5263 | 0.4881 | | NA | | NA | | | NA | NA | | NA | | NA | | NA | NA |
| Red junglefowl | | **0.0019** | **0.0146** | | 0.4058 | | NA | | | NA | NA | | NA | | NA | | NA | NA |
| Ukokkei | | **0.0019** | **0.0045** | | 0.3716 | | 0.7598 | | | NA | NA | | NA | | NA | | NA | NA |
| Polish | | **0.0015** | **0.0088** | | 0.1139 | | **0.0189** | | | 0.1459 | NA | | NA | | NA | | NA | NA |
| White leghorn | | 0.0573 | 0.0538 | | 0.5116 | | 0.6200 | | | 0.9189 | 0.7133 | | NA | | NA | | NA | NA |
| Appenzeller Spitzhaubenhuhn | | **0.0031** | **0.0095** | | 0.1511 | | 0.0538 | | | 0.2038 | 1.0000 | | 0.7874 | | NA | | NA | NA |
| Araucana | | **0.0001** | **0.0017** | | **0.0437** | | **0.0104** | | | 0.2038 | 0.7714 | | 0.7145 | | 0.6145 | | NA | NA |
| Cochin (standard) | | **0.0095** | **0.0294** | | 0.1897 | | **0.0045** | | | 0.1292 | 0.1871 | | 0.2038 | | 0.3716 | | 0.2420 | NA |
| Shamo | | **0.0017** | **0.0104** | | 0.1728 | | 0.2420 | | | 0.4354 | 0.2847 | | 1.0000 | | 0.4058 | | 0.4140 | 0.0606 |
|  | |  |  | |  | |  | | |  |  | |  | |  | |  |  |
| **Carpometacarpus** | | | | | | | | | | | | | | | | | | |
|  | | Chabo | Bantam | | Cochin (dwarf) | | Red junglefowl | | | Ukokkei | Polish | | White leghorn | | Appenzeller Spitzhaubenhuhn | | Araucana | Cochin (standard) |
| Bantam | | 0.8731 | NA | | NA | | NA | | | NA | NA | | NA | | NA | | NA | NA |
| Cochin (dwarf) | | 0.8731 | 0.9135 | | NA | | NA | | | NA | NA | | NA | | NA | | NA | NA |
| Red junglefowl | | 0.8731 | 0.9971 | | 0.5946 | | NA | | | NA | NA | | NA | | NA | | NA | NA |
| Ukokkei | | 0.5946 | 0.5244 | | 1.0000 | | 0.3466 | | | NA | NA | | NA | | NA | | NA | NA |
| Polish | | 0.5946 | 0.2350 | | 0.7171 | | 0.2350 | | | 0.1573 | NA | | NA | | NA | | NA | NA |
| White leghorn | | 0.5946 | 0.5992 | | 1.0000 | | 0.4011 | | | 0.8731 | 0.5440 | | NA | | NA | | NA | NA |
| Appenzeller Spitzhaubenhuhn | | 0.8596 | 0.8731 | | 0.5946 | | 0.8525 | | | 0.5946 | 0.6044 | | 0.5820 | | NA | | NA | NA |
| Araucana | | 0.2350 | 0.1857 | | 0.4239 | | 0.1635 | | | 0.0519 | 0.6422 | | 0.4483 | | 0.4542 | | NA | NA |
| Cochin (standard) | | 0.5121 | 0.6463 | | 0.5946 | | 0.5244 | | | 0.7336 | 0.5946 | | 0.5946 | | 0.5946 | | 0.3806 | NA |
| Shamo | | 0.0971 | 0.0679 | | 0.3333 | | 0.1635 | | | 0.0519 | 0.2344 | | 0.5121 | | 0.4011 | | 0.7438 | 0.1667 |
|  | |  |  | |  | |  | | |  |  | |  | |  | |  |  |
| **Femur** | | | | | | | | | | | | | | | | | | |
|  | | Chabo | Bantam | | Cochin (dwarf) | | Red junglefowl | | | Ukokkei | Polish | | White leghorn | | Appenzeller Spitzhaubenhuhn | | Araucana | Cochin (standard) |
| Bantam | | **0.0028** | NA | | NA | | NA | | | NA | NA | | NA | | NA | | NA | NA |
| Cochin (dwarf) | | **0.0345** | 0.3407 | | NA | | NA | | | NA | NA | | NA | | NA | | NA | NA |
| Red junglefowl | | **0.0000** | **0.0014** | | 0.1752 | | NA | | | NA | NA | | NA | | NA | | NA | NA |
| Ukokkei | | **0.0000** | **0.0182** | | 0.6219 | | 0.0953 | | | NA | NA | | NA | | NA | | NA | NA |
| Polish | | **0.0002** | **0.0014** | | 0.0546 | | 0.3597 | | | **0.0028** | NA | | NA | | NA | | NA | NA |
| White leghorn | | **0.0028** | 0.1491 | | 0.5238 | | 0.6459 | | | 0.2537 | 0.8205 | | NA | | NA | | NA | NA |
| Appenzeller Spitzhaubenhuhn | | **0.0007** | **0.0028** | | 0.0755 | | 0.1514 | | | **0.0157** | 0.8221 | | 0.8032 | | NA | | NA | NA |
| Araucana | | **0.0000** | **0.0000** | | **0.0109** | | 0.2847 | | | **0.0014** | 0.5974 | | 0.9740 | | 0.4737 | | NA | NA |
| Cochin (standard) | | **0.0067** | **0.0183** | | 0.1774 | | 0.7202 | | | 0.0593 | 0.5238 | | 0.7202 | | 0.5238 | | 1.0000 | NA |
| Shamo | | **0.0000** | **0.0004** | | **0.0303** | | 0.6427 | | | **0.0028** | 0.1854 | | 0.4116 | | 0.4116 | | 0.7242 | 0.8205 |
|  | |  |  | |  | |  | | |  |  | |  | |  | |  |  |
| **Tibiotarsus** | | | | | | | | | | | | | | | | | | |
|  | | Chabo | Bantam | | Cochin (dwarf) | | Red junglefowl | | | Ukokkei | Polish | | White leghorn | | Appenzeller Spitzhaubenhuhn | | Araucana | Cochin (standard) |
| Bantam | | 0.0796 | NA | | NA | | NA | | | NA | NA | | NA | | NA | | NA | NA |
| Cochin (dwarf) | | 0.3169 | 0.8795 | | NA | | NA | | | NA | NA | | NA | | NA | | NA | NA |
| Red junglefowl | | **0.0162** | 0.9208 | | 0.6516 | | NA | | | NA | NA | | NA | | NA | | NA | NA |
| Ukokkei | | 0.2556 | 0.4948 | | 0.5252 | | 0.2556 | | | NA | NA | | NA | | NA | | NA | NA |
| Polish | | 0.5933 | 0.1035 | | 0.2015 | | 0.0827 | | | 0.2015 | NA | | NA | | NA | | NA | NA |
| White leghorn | | 0.3905 | 0.8523 | | 0.8523 | | 0.8527 | | | 0.9087 | 0.2556 | | NA | | NA | | NA | NA |
| Appenzeller Spitzhaubenhuhn | | 0.2556 | 0.8523 | | 0.9087 | | 0.9061 | | | 0.5252 | 0.2556 | | 0.8258 | | NA | | NA | NA |
| Araucana | | **0.0043** | 0.2556 | | 0.9519 | | 0.1714 | | | **0.0242** | **0.0162** | | 0.3962 | | 0.9087 | | NA | NA |
| Cochin (standard) | | 0.3169 | 0.9724 | | 1.0000 | | 0.9087 | | | 0.6516 | 0.2619 | | 0.8523 | | 1.0000 | | 0.8795 | NA |
| Shamo | | 0.9501 | 0.1714 | | 0.3951 | | 0.1035 | | | 0.3951 | 0.5750 | | 0.5185 | | 0.3169 | | **0.0242** | 0.3169 |
|  | |  |  | |  | |  | | |  |  | |  | |  | |  |  |
| **Tarsometatarsus** | | | | | | | | | | | | | | | | | | |
|  | | Chabo | Bantam | | Cochin (dwarf) | | Red junglefowl | | | Ukokkei | Polish | | White leghorn | | Appenzeller Spitzhaubenhuhn | | Araucana | Cochin (standard) |
| Bantam | | **0.0354** | NA | | NA | | NA | | | NA | NA | | NA | | NA | | NA | NA |
| Cochin (dwarf) | | 0.0826 | 0.7615 | | NA | | NA | | | NA | NA | | NA | | NA | | NA | NA |
| Red junglefowl | | **0.0000** | **0.0056** | | 0.2648 | | NA | | | NA | NA | | NA | | NA | | NA | NA |
| Ukokkei | | **0.0000** | **0.0214** | | 0.3004 | | 0.3693 | | | NA | NA | | NA | | NA | | NA | NA |
| Polish | | **0.0003** | **0.0014** | | 0.0635 | | **0.0101** | | | **0.0006** | NA | | NA | | NA | | NA | NA |
| White leghorn | | **0.0227** | 0.1879 | | 0.5000 | | 0.4615 | | | 0.2537 | 0.4615 | | NA | | NA | | NA | NA |
| Appenzeller Spitzhaubenhuhn | | **0.0012** | **0.0056** | | 0.1511 | | 0.2814 | | | 0.1206 | 0.7589 | | 0.9048 | | NA | | NA | NA |
| Araucana | | **0.0000** | **0.0012** | | 0.1903 | | 0.7623 | | | 0.1511 | **0.0044** | | 0.3460 | | 0.3004 | | NA | NA |
| Cochin (standard) | | **0.0142** | 0.1511 | | 0.3004 | | 0.8711 | | | 0.5212 | 0.1139 | | 0.5000 | | 0.6832 | | 0.8302 | NA |
| Shamo | | **0.0000** | **0.0004** | | 0.0635 | | 0.1717 | | | **0.0056** | 0.1541 | | 0.7615 | | 0.7659 | | 0.1511 | 0.7376 |
